# Identification of genomic regions and candidate genes of functional importance for gastrointestinal parasite resistance traits in Djallonké sheep of Burkina Faso

**DOI:** 10.5194/aab-62-313-2019

**Published:** 2019-06-05

**Authors:** Isabel Álvarez, Iván Fernández, Albert Soudré, Amadou Traoré, Lucía Pérez-Pardal, Moumouni Sanou, Stephane A. R. Tapsoba, Nuria A. Menéndez-Arias, Félix Goyache

**Affiliations:** 1 SERIDA Deva., Camino de Rioseco 1225, 33394 Gijón (Asturias), Spain; 2 Université de Koudougou, BP 376 Koudougou, Burkina Faso, Burkina Faso; 3 Institut de l'Environnement et Recherches Agricoles (INERA), 04 BP 8645 Ouagadougou 04, Burkina Faso; 4 CIBIO-InBIO, Universidade do Porto, 4485-661 Vairão, Portugal

## Abstract

A total of 184 Djallonké lambs from Burkina Faso with phenotypes for packed-cell volume (PCV), log-transformed fecal egg count (lnFEC), and FAffa MAlan CHArt (FAMACHA©) eye scores were typed with the OvineSNP50 BeadChip of Illumina to contribute to the knowledge of the genetic basis of gastrointestinal (GIN) parasite resistance in sheep. Association analysis identified a total of 22 single-nucleotide polymorphisms (SNPs) related with PCV (6 SNPs), lnFEC (7), and FAMACHA scores (9) distributed among 14 *Ovis aries* chromosomes (OAR). The identified SNPs accounted for 18.76 % of the phenotypic variance for PCV, 21.24 % for lnFEC, and 34.38 % for FAMACHA scores. Analyses pointed out the importance of OAR2 for PCV, OAR3 for FAMACHA scores, and OAR6 for lnFEC. The 125 kb regions surrounding the identified SNPs overlapped with seven previously reported quantitative trait loci (QTLs) for the traits analyzed in the current work. The only chromosome harboring markers associated with the three traits studied was OAR2. In agreement with the literature, two different chromosomal areas on OAR2 can play a major role in the traits studied. Gene-annotation enrichment analysis allowed us to identify a total of 34 potential candidate genes for PCV (6 genes), lnFEC (4), and FAMACHA scores (24). Annotation analysis allowed us to identify one functional term cluster with a significant enrichment score (1.302). The cluster included five genes (*TRIB3*, *CDK4*, *CSNK2A1*, *MARK1*, and *SPATA5*) involved in immunity-related and cell-proliferation processes. Furthermore, this research suggests that the *MBL2* gene can underlie a previously reported QTL for immunoglobulin A levels on OAR22 and confirms the importance of genes involved in growth and size (such as the *ADAMTS17* gene on OAR18) for GIN resistance traits. Since association studies for the ascertainment of the genetic basis of GIN resistance may be affected by genotype–environment interactions, obtaining information from local sheep populations managed in harsh environments contributes to the identification of novel genomic areas of functional importance for GIN resistance for that trait.

## 1 Introduction

Gastrointestinal (GIN) parasite infections are a major obstacle for sustainable small-ruminant production due to their detrimental effects upon food digestion and utilization, skeletal and muscular development, fertility, wool and milk production, mortality rates, and antiparasitic expenditure (Jackson et al., 2009). It is of particular importance to tropical production systems in which the costs of disease have been estimated as 35 % to 50 % of turnover in market-oriented terms (Bishop, 2012). Furthermore, there is an increasing interest to limit the use of anthelmintic medicine to avoid the emergence of negative consequences for human health such as the emergence of resistant strains of parasites and the presence of drug residues in animal products (Jackson et al., 2009; Stear et al., 2007).

Among different alternatives, the implementation of selection schemes aiming to increase resistance to GIN infections has been proposed (Bishop and Morris, 2007). However, recording indirect indicators of GIN resistance (namely fecal egg count), immune response to infection (such as antibody (IgA, IgG, or IgM) levels), or the impact of infection (such as anemia, e.g., packed-cell volume) is difficult, frequently invasive, and dependent on the existence of an efficient performance recording system. Furthermore, most heritability estimates reported for traits related to GIN resistance varied from moderate to low (Safari et al., 2005; Gutiérrez-Gil et al., 2010; Goldberg et al., 2012). Therefore, selection for such a complex phenotype is costly and time consuming. These requirements are not frequently met in the small-ruminant industry, particularly in low-income smallholder systems which are predominant in developing countries (Zvinorova et al., 2016).

Molecular markers could be used to enhance the genetic response to selection for GIN resistance. However, the identification of candidate genes for GIN resistance has remained elusive. Quantitative trait loci (QTL) studies, using microsatellite markers, identified multiple significantly associated loci, scattered throughout the ovine genome, with little regions of overlap among the results reported (Gutiérrez-Gil et al., 2009b; Marshall et al., 2009; Sallé et al., 2012). The availability of medium-density single-nucleotide polymorphism (SNP) chips has allowed us to refine the information obtained from previous QTL studies (Atlija et al., 2016; Benavides et al., 2015; Berton et al., 2017; Pickering et al., 2015). However, gathering information from different sheep populations is still needed to reach a deeper understanding of the genetic architecture of GIN resistance. Different breeds may vary in ability to live and produce in environments highly contaminated with GIN larvae (Amarante et al., 2004; Rocha et al., 2004). Furthermore, genotype–environment interactions may be expected if animals are managed in environments that differ in terms of the extent of parasite challenge or the quality of available nutrition (Bishop, 2012). Therefore, information about GIN resistance from a broad array of sheep populations is still a pending issue.

Recently, a field trial designed to ascertain the factors affecting gastrointestinal parasite resistance in Djallonké (West African Dwarf) lambs of Burkina Faso was performed (Traoré et al., 2017). This information was partially used to estimate genetic parameters and estimated breeding values (EBVs) for GIN resistance traits using pedigree-free animal models (Álvarez et al., 2018). Within this context, a genome-wide association study (GWAS) was performed in the Djallonké sheep of Burkina Faso to contribute to the knowledge of the genetic basis of GIN resistance in sheep.

## 2 Material and methods

### 2.1 Data and estimated breeding values

Performance data were obtained from a trial designed to assess environmental factors affecting gastrointestinal parasite resistance in Djallonké sheep of Burkina Faso. Morphological and genetic description of this sheep population can be found in Traoré et al. (2008) and Álvarez et al. (2009, 2012). Data were obtained from (a) a field trial, involving 434 lambs, carried out in the surroundings of Mangodara (Comoé province) located in the southern Sudan to Guinea savannah region of Burkina Faso during the rainy season 2014 (Traoré et al., 2017) and from (b) 19 lambs sampled in Dédougou (Mouhoun province), southwestern Burkina Faso, and submitted simultaneously to the same protocol at the facilities of the Kamboinsé station of the INERA (Álvarez et al., 2018) near Ouagadougou (central Sudan to Sahel savannah region of Burkina Faso). Climate and sheep management in southern Burkina Faso was previously described in Álvarez et al. (2009) and Traoré et al. (2017). Briefly, the humid southern sudan to Guinea savannah region of Burkina Faso covers from latitude 9${}^{\circ }$30${}^{\prime }$ N to latitude 11${}^{\circ }$30${}^{\prime }$ N and has annual rainfall higher than 900 mm; animals traditionally graze in communal native pasture with no supplementation for 8 to 14 h d${}^{-1}$ during the rainy season. Grazing time is not restricted during the dry season.

As reported by Traoré et al. (2017), lambs were dewormed with levamisol, following the manufacturer's recommendations, at a dose of 7.5 mg kg${}^{-1}$ (Traoré et al., 2017). Individuals were assessed at 28 and 35 d after deworming for body weight, packed-cell volume (PCV), fecal egg count (FEC), and FAffa MAlan CHArt (FAMACHA©) eye scores. FEC scores were log-transformed as lnFEC = ln(FEC + 25). Before and after deworming, individuals were exposed to natural infection with gastrointestinal nematodes. Deworming and sampling were performed by veterinary practitioners with the permission and in the presence of the owners.

Up to 271 individuals (252 sampled in Mangodara) yielded blood samples (Álvarez et al., 2018) useful for DNA extraction using standard procedures (Sambrook et al., 1989). As described in Álvarez et al. (2018), 29 microsatellites were typed on all samples (Automatic Sequencer ABI 310, Applied Biosystems, Barcelona) to infer an artificial pedigree of 10 discrete generations using the software MOLCOAN (Fernández and Toro, 2006; Cervantes et al., 2011). The algorithm implemented in MOLCOAN maximizes the correlation between the coancestry molecular matrix, given the data, and the genealogical coancestry matrix built from the artificial pedigree. Performance data and the artificial pedigree were used to estimate genetic parameters and EBVs for PCV, lnFEC, and FAMACHA scores under a Bayesian approach using the program TM (Threshold Model; http://snp.toulouse.inra.fr/~alegarra/, last access: 15 May 2015). Briefly, models fitted for analyses (Álvarez et al., 2018) included the following fixed effects: (a) contemporary group (10 levels) formed by the individuals of the same sex and the same age (in months) assessed in either the field trial (Mangodara) or the station trial (Kamboinsé), (b) days after deworming (two levels: days 28 and 35), and (c) body weight at the moment of assessment as a linear covariate. Models also used the following random effects: (a) permanent environment associated to the individual and (b) the additive genetic effect. FAMACHA scores were treated assuming a threshold model. EBVs estimated for PCV, lnFEC, and FAMACHA scores using univariate models were further used as phenotypes for association analyses.

The use of EBVs as phenotypes for genome-wide association studies is not recommended due to increase of type I error and deflated estimates of the QTL effect (Ekine et al., 2014). The use of “yield deviations” (Atlija et al., 2016) estimated by adjusting performance for major environmental factors affecting phenotypes is a widely used alternative. However, the adjustment of performance for the main environmental effects in our data was difficult: although the householder of the individuals was recorded, the identification of the communal grazing unit (the actual management unit; Traoré et al., 2017) to which the animals belonged was not known; actual pedigrees were not known and major effects such the membership to a given litter of the family effect (Ekine et al., 2014) could not be included in the model to be fitted; and, finally, the actual age was not known but approximated by examining dentition. In such a scenario, we opted to use the EBVs estimated in Álvarez et al. (2018). Note that the EBVs used are basically the phenotypes corrected for the influence of a molecular relatedness matrix constructed using microsatellites.

### 2.2 SNP genotyping, quality control, and structuring

Only 184 DNA samples (64 males and 120 females) from both the Mangodara (166) and the Dédougou (18) trials had enough quality to be analyzed using the OvineSNP50 BeadChip following standard protocols (http://www.illumina.com, last access: 12 March 2019). The software GenomeStudio (Illumina Inc., San Diego, CA) was used to generate standard .ped and .map files. Sample and marker-based quality control measures were performed using the software PLINK v 1.07 (Purcell et al., 2007). A GenCall score cutoff of 0.15 and an average sample call rate of 99 % were considered. All unmapped SNPs, those mapping to sexual chromosomes, SNPs with a genotyping rate lower than 90 %, or those below a minor allele frequency (MAF) threshold of 0.05 were removed. To avoid departures from Hardy–Weinberg proportions due to genotyping errors, SNPs that did not pass Hardy–Weinberg test for P≤0.001 were removed as well. A total of 46 977 SNPs located on the 26 ovine autosomes passed the quality control for the population of 184 Djallonké lambs.

The software PLINK v 1.07 (Purcell et al., 2007) and Arlequin 3.5 (Excoffier and Lischer, 2010) were used to compute parameters characterizing genetic diversity of dataset (expected homozygosity and $F_{\mathrm{ST}}$ values).

A clustering analysis was carried out using the software Admixture v 1.23 (Alexander et al., 2009; Alexander and Lange, 2011), which calculates maximum likelihood estimates of individual ancestries based on data provided by multiple loci. Analyses were conducted for 1≤K≤10, K being the number of clusters given in the data. The optimal number of clusters was determined via cross-validation as the value of K exhibiting the lower cross-validation error compared to other K values. The dataset was divided into 5 folders for each K. Folders were sequentially used as test sets while the other four were used for training.

### 2.3 Marker association analysis

SNPs associated with EBVs estimated for PCV, lnFEC, and FAMACHA scores were identified using the polygenic-background-control-based least angle regression plus empirical Bayes method (pLARmEB; Zhang et al., 2017) implemented in the mrMLM software package (Wang et al., 2016) of R (http://CRAN.R-project.org/, last access: 15 May 2019). The pLARmEB method integrates a least angle regression with empirical Bayes method to perform multi-locus genome-wide association analysis (GWAS) under polygenic background control using an algorithm of model transformation that whitens the relationship matrix of the polygenic matrix K and environmental noise. Markers on one chromosome are simultaneously analyzed by fitting a multi-locus model and least angle regression is used to select the most potentially associated SNPs. In turn, the markers on the other chromosomes are used to calculate a kinship matrix as a polygenic background control. The selected SNPs in the multi-locus model are further analyzed for their association with the trait by empirical Bayes and likelihood ratio test. Following the recommendations of the authors, no Bonferroni correction was applied for false discovery rate but a critical logarithm of the odds (LOD) score higher than 2. Furthermore, the pLARmEB method was performed by fitting 50 as the number of potentially associated variables to be selected and including population structuring (individual ancestry fractions estimated using the software Admixture v 1.23).

### 2.4 Functional characterization of the candidate regions

Candidate genes were considered if their boundaries fell within 125 kb upstream or downstream of the significant SNPs (Atlija et al., 2016). Protein-coding genes found within the candidate regions were retrieved from the Ensembl Genes 91 database, based on the Oar v3.1 ovine reference genome (http://www.livestockgenomics.csiro.au/sheep/oar3.1.php, last access: 1 March 2019) using the BioMart tool (Kinsella et al., 2011). All the identified genes were processed using the functional annotation tool implemented in DAVID Bioinformatics Resources 6.8 (Huang et al., 2009) to determine enriched functional terms. An enrichment score of 1.3, which is equivalent to the Fisher exact test P value of 0.05, was used as a threshold to define the significantly enriched functional terms in comparison to the whole ovine reference genome background.

The ovine QTLs previously mapped on the ovine Genome Assembly Oar_v3.1 were downloaded from the sheep QTL database (https://www.animalgenome.org/cgi-bin/QTLdb/OA/index, last access: 15 December 2018). The intersectBed function of the BedTools software (Quinlan and Hall, 2010) was used to overlap these QTLs with the identified candidate regions.

**Figure 1:**
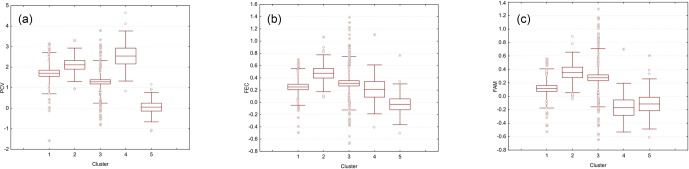
Boxplots illustrating the variation in the EBVs for PCV **(a)**, lnFEC **(b)**, and FAMACHA scores **(c)** used as phenotypes for association analyses grouped by each of the five Clusters identified using the software Admixture v 1.23. The box represents the range that contains the values within the limits of the standard error of the mean, the line within the box indicating the mean value. The whiskers are the lines that extend from the box to the standard deviation, excluding outliers and extreme values. Outliers, which are represented by circles, are values that are 1.5–3 standard error lengths from the upper or lower edge of the box. Extreme values, which fall outside the standard deviation limits, are represented by asterisks. Clusters from 1 to 5 included 45, 15, 100, 45, and 14 individuals, respectively.

## 3 Results

### 3.1 Description of the variability in the dataset

Mean (and SD) of the EBVs estimated for PCV, lnFEC, and FAMACHA scores were 1.430 (1.126), 0.278 (0.378), and 0.189 (0.408), respectively. The heritabilities estimated using the same models fitted for estimating the EBVs used was 0.240±0.114 for PCV, 0.225±0.088 for lnFEC, and 0.352±0.157 for FAMACHA scores (Álvarez et al., 2018). Observed homozygosity computed for the whole dataset was 0.386 (0.044). The results of the admixture analysis informed us that the lowest cross-validation error was at K=5 (Fig. S1 in the Supplement). Cluster membership varied from 100 individuals for Cluster 3 to 14 individual for Cluster 4 (Fig. 1). The individuals classified into each cluster showed large variability for the phenotypes to be used in association analyses (Fig. 1). Parameters characterizing phenotypic and genetic diversity of the analyzed population by cluster are given in Table S1 in the Supplement. Except for the pair 2–4 ($F_{\mathrm{ST}}=0.017$), the clusters identified in the population studied had genetic differences consistent with population structuring, particularly for the pairs involving Cluster 1 ($F_{\mathrm{ST}}$ values varying from 0.075 to 0.164) and the pair of clusters 3–5 ($F_{\mathrm{ST}}=0.144$).

**Table 1:** List of SNPs significantly associated to performance (LOD scores >2) for packed-cell volume (PCV), log-transformed fecal egg count (lnFEC), and FAMACHA scores. Names of the genes identified within the 125 kb regions surrounding the SNPs identified are also given. A full description of these genes is given in Table S2.

Trait	SNP	OAR${}^{1}$	Position${}^{2}$	QTN effect	LOD score	P value	Proportion${}^{3}$	MAF${}^{4}$	Gene name
PCV									
1	OAR1_55820164.1	1	55 820 164	0.267	5.153	$1.11\times 10^{-2}$	5.892	0.380	
2	s23524.1	2	49 877 948	-0.326	4.919	$1.94\times 10^{-2}$	7.373	0.272	*TMOD1; TDRD7*
3	OAR2_117867801.1	2	117 867 801	0.194	2.917	$2.00\times 10^{-4}$	2.838	0.315	*MFSD6; INPP1; HIBCH; C2H2orf88*
4	OAR8_16568165.1	8	16 568 165	0.014	2.519	$7.00\times 10^{-4}$	0.017	0.435	
5	OAR15_88875909.1	15	88 875 909	-0.113	2.327	$1.10\times 10^{-3}$	0.988	0.332	
6	OAR18_43101149.1	18	43 101 149	0.140	3.551	$5.25\times 10^{-1}$	1.647	0.419	
lnFEC									
7	OAR2_140684314.1	2	140 684 314	0.080	4.244	$9.84\times 10^{-2}$	3.901	0.342	
8	s16493.1	6	117 462 479	-0.098	6.646	$3.16\times 10^{-4}$	6.579	0.489	
9	s43307.1	7	7 323 364	-0.050	3.314	$9.35\times 10^{-1}$	1.399	0.283	*SV2C; IQGAP2*
10	OAR8_8982479.1	8	8 982 479	-0.045	2.186	$1.50\times 10^{-3}$	1.096	0.277	
11	OAR15_2525103.1	15	2 525 103	0.109	5.709	$2.93\times 10^{-3}$	8.102	0.489	
12	OAR17_34531123_X.1	17	34 531 124	0.017	3.151	$1.00\times 10^{-4}$	0.194	0.451	*SPATA5; NUDT6*
13	s43852.1	19	41 182 624	-0.010	2.340	$1.00\times 10^{-3}$	0.068	0.478	
FAMACHA									
14	OAR2_64824262.1	2	64 824 262	-0.082	4.544	$4.78\times 10^{-2}$	4.752	0.386	
15	OAR3_77774489.1	3	77 774 489	-0.097	4.331	$7.97\times 10^{-2}$	6.480	0.353	*ATP6V1E2; TMEM247; EPAS1*
16	OAR3_161498140.1	3	161 498 140	0.076	3.679	$3.85\times 10^{-1}$	4.242	0.451	*ATP23; CTDSP2; AVIL; TSFM; METTL21B;*
									*METTL1; LOC101116039; MARCH9; CDK4; TSPAN31*
17	OAR12_22189408.1	12	22 189 408	-0.073	4.710	$3.20\times 10^{-3}$	3.572	0.342	*MARK1; C12H1orf115*
18	s32476.1	13	59 926 032	-0.063	5.430	$5.72\times 10^{-3}$	2.937	0.424	*CSNK2A1; TBC1D20; RBCK1; TRIB3; NRSN2;*
									*ZCCHC3; C13H20orf96*
19	s09612.1	13	76 546 482	0.086	4.752	$2.90\times 10^{-2}$	5.554	0.473	
20	OAR18_5508052_X.1	18	5 508 053	-0.058	3.510	$5.81\times 10^{-1}$	2.499	0.462	*ADAMTS17*
21	OAR22_6293170.1	22	6 293 170	-0.068	4.817	$2.48\times 10^{-3}$	3.413	0.462	*MBL2*
22	OARX_107840506.1	23	107 840 506	-0.069	2.594	$5.00\times 10^{-4}$	0.927	0.071	

${}^{1}$ *Ovis aries* chromosome on which the SNP is located. ${}^{2}$ SNP position on the chromosome (in bp). ${}^{3}$ Proportion (in percentage) of phenotypic variance explained by the putative quantitative trait nucleotide (QTN). Estimates of error variance and total phenotypic variance were 0.740 and 1.267 for PCV, 0.110,and 0.159 for lnFEC, and 0.075 and 0.166 for FAMACHA scores, respectively. ${}^{4}$ Minor allele frequency.

### 3.2 Association analysis and functional candidate genes

The pLARmEB algorithm identified a total of 22 SNPs (Table 1) associated with PCV (6), lnFEC (7), and FAMACHA scores (9) distributed among 14 *Ovis aries* chromosomes (OAR). SNPs associated with the three traits analyzed were only found on OAR2. SNPs associated with PCV and lnFEC were found on OAR8, while SNPs associated with PCV and FAMACHA scores were found on OAR18. The identified SNPs accounted for 18.76 % of the phenotypic variance for PCV (varying from 0.02 % to 7.37 %), 21.24 % for lnFEC (from 0.07 % to 8.10 %), and 34.38 % (from 0.93 % to 6.48 %) for FAMACHA score.

Gene-annotation enrichment analysis allowed us to identify a total of 34 potential candidate genes in the 125 kb upstream and downstream regions surrounding the SNPs associated with performance for PCV (6 genes), lnFEC (4 genes) and FAMACHA scores (24 genes; Table 1). Twelve out of 22 regions associated to the identified SNPs (four for PCV, five for lnFEC and three for FAMACHA scores) did not span candidate genes based on the Oar v3.1 ovine reference genome (Table 1). This set included the two SNP regions associated with PCV and lnFEC on OAR8.

**Table 2:** Significantly enriched functional term cluster (enrichment score = 1.302) following DAVID analysis for genes identified within the 125 kb regions flanking the SNPs associated with performance for PCV, lnFEC and FAMACHA scores in Djallonké sheep.

Category	Term	Count	P value	Fold	Candidate genes
				enrichment	
SMART	SM00220:S_TKc	4	0.017	6.624	ENSOARG00000019129 (*TRIB3*) ENSOARG00000005320 (*CDK4*) ENSOARG00000019053 (*CSNK2A1*) ENSOARG00000012956 (*MARK1*)
INTERPRO	IPR000719:Protein kinase, catalytic domain	4	0.042	4.926	ENSOARG00000019129 (*TRIB3*) ENSOARG00000005320 (*CDK4*) ENSOARG00000019053 (*CSNK2A1*) ENSOARG00000012956 (*MARK1*)
INTERPRO	IPR011009:Protein kinase-like domain	4	0.052	4.515	ENSOARG00000019129 (*TRIB3*) ENSOARG00000005320 (*CDK4*) ENSOARG00000019053 (*CSNK2A1*) ENSOARG00000012956 (*MARK1*)
GOTERM_MF_DIRECT	GO:0005524∼ATP binding	5	0.164	2.196	ENSOARG00000019129 (*TRIB3*) ENSOARG00000005320 (*CDK4*) ENSOARG00000017222 (*SPATA5*) ENSOARG00000019053 (*CSNK2A1*) ENSOARG00000012956 (*MARK1*)

A full description of the 34 potential candidate genes is given in Table S2. Functional annotation informed that these genes were associated with different biological functions, including spermatogenesis and transmembrane and kinase activities (Table S2). Annotation analysis carried out on these 34 genes identified three functional term clusters (Table S3). However, only one of them had an enrichment score higher than 1.3 (Table 2). The cluster included five genes (*TRIB3*, *CDK4*, *CSNK2A1*, *MARK1*, and *SPATA5*) involved in immunity-related and cell-proliferation processes (Table S2).

### 3.3 Correspondence with previously reported QTLs in sheep

A total of 102 QTLs previously reported on 10 different chromosomes in domestic sheep intersected with the 125 kb regions surrounding 19 out of the 22 SNPs associated with PCV, lnFEC, and FAMACHA EBVs in Djallonké sheep (Table S4). Only seven of these QTLs were directly related with the traits analyzed in the current work: (a) a QTL for *Trichostrongylus* adult and larva count on OAR2 (Crawford et al., 2006) overlapped with the regions surrounding SNPs OAR2_117867801.1 and OAR2_64824262.1, associated with PCV and FAMACHA scores, respectively (Table 2); (b) on OAR8 one QTL for FEC (Atlija et al., 2016) overlapped with SNP OAR8_8982479.1 (lnFEC) and two QTLs for *Trichostrongylus* adult and larva count reported by Crawford et al. (2006) overlapped the regions surrounding that SNP and marker OAR8_16568165.1 (PCV); (c) on OAR13 a QTL for FEC (Silva et al., 2012) overlapped with the region surrounding SNP s09612.1 (FAMACHA scores); (d) on OAR18 a QTL for hematocrit (Silva et al., 2012) overlapped with the region on which SNP OAR18_5508052_X.1 (FAMACHA scores) was located; and finally (e) a QTL for immunoglobulin A level reported by Atlija et al. (2016) on OAR22 overlapped with the region surrounding marker OAR22_6293170.1 (FAMACHA scores).

The other QTLs listed in Table S4 were associated with various traits, namely related to weight and growth (Cavanagh et al., 2010; Fullard et al., 2006; Matika et al., 2016; McRae et al., 2005; Roldan et al., 2010), fleece (Ponz et al., 2001; Allain et al., 2006), carcass (Cavanagh et al., 2010; Matika et al., 2016), meat (Johnson et al., 2005; Karamichou et al., 2006), and dairy traits (Gutiérrez-Gil et al., 2008, 2009a, 2011; García-Gámez et al., 2013; Mateescu and Thonney, 2010; Raadsma et al., 2009).

## 4 Discussion

Although most of the analyzed individuals were sampled from a single local population, the available dataset gathered a noticeable diversity (Fig. 1). Djallonké sheep is a basically unselected population and, therefore, wide differences among individuals' performance are expected. Using microsatellites, Álvarez et al. (2018) also identified some genetic structuring in the current population. These authors considered structuring a consequence of different unexpected founder events. In Burkina Faso, management of Djallonké sheep flocks is carried out under very traditional conditions (Traoré et al., 2017) with no supervised matings. In any case, Álvarez et al. (2018) explained that individuals classified into different genetic clusters were randomly distributed among the comparison groups used in the model fitted for the estimation of the EBVs. Therefore, the EBVs used here as phenotypes for association analyses are not likely to be biased. Overall, the variability included in the current dataset can be considered useful for the intended purposes.

### 4.1 Consistency with previous analyses

Microsatellite-based QTL approaches identified a very wide number of chromosome regions with small to moderate effects associated with GIN resistance in sheep (Gutiérrez-Gil et al., 2009b; Sallé et al., 2012). In any case, analyses established a consensus about the importance of chromosomal regions surrounding the interferon gamma (*IFNG*) locus (OAR3; positions from 151, 527, 165 to 151, 535, 188) and within or adjacent to the major histocompatibility complex (MHC) region (OAR20; positions from 12 326 227 to 40 705 933) (Bishop, 2012; Zvinorova et al., 2016). GWAS refined the information provided by QTL analyses: the putative importance of OAR3 and OAR20 regions on GIN resistance was confirmed but some regions on OAR1 and OAR6 tended to appear consistently across studies (Benavides et al., 2016). In addition, Sweeney et al. (2016) suggested that chromosomal regions of OAR14, probably linked to the toll-like receptor *IRF3* gene (Matika et al., 2016), could be important for resistance to GIN infection.

It is well known that there are discrepancies between association analyses (using either QTL or GWAS approaches) for GIN resistance in sheep. Causes of such a lack of consistent results among studies are (a) the age of individuals used, with key pathways preventing primary parasite infections (in lambs) probably being different from those involved in subsequent infections in adult sheep (Gutiérrez-Gil et al., 2010); (b) GIN type challenge, with some marker regions probably being exclusive to particular parasite species (Benavides et al., 2016); (c) environmental (e.g., nutritional or GIN exposition) differences, which may interact with differences in genetic background among the sheep populations studied (Bishop, 2012); and (d) the quantitative nature of host resistance, probably determined by multiple genes with varied effects rather than by a limited number of major genes (Benavides et al., 2016). Furthermore, a non-negligible practical concern is that GIN resistance studies assume that all animals have been exposed during the same time to the same level of infective larvae in the pasture. While this assumption is almost impossible to fulfill in a grazing environment, data coming from field trials, such as that implemented in Djallonké sheep (Traoré et al., 2017; Álvarez et al., 2018), can minimize some environmental factors affecting results.

The current work did not identify significant SNPs associated to GIN resistance neither on OAR14 nor on OAR20. Although information on the importance of OAR14 is less apparent, many studies have reported association between OAR20 and for GIN resistance traits (Sweeney et al., 2016; Benavides et al., 2016). However, exceptions are not rare: several QTL and GWAS studies have not yielded associations between such performance and the areas surrounding the MHC region (Beh et al., 2002; Gutiérrez-Gil et al., 2009b; Pickering et al., 2015; Berton et al., 2017). Furthermore, no clear selection signatures have been reported in this region of OAR20 using medium-density SNP Chips (McRae et al., 2014). The highly polymorphic nature of the MHC region is likely to make it difficult to identify SNPs useful for selection for GIN resistance (Sweeney et al., 2016).

In any case, the current research relatively agrees with the importance of OAR3 and OAR6 for GIN resistance traits.

Only performance for FAMACHA scores was associated with markers located on OAR3. The two SNPs identified (explaining a total of 10.72 % of the phenotypic variance for this trait) were located in distant chromosomal areas with marker OAR3_161498140.1 located in the relative vicinity of the *INFG* locus (Table 1). This scenario is consistent with expectations suggesting that several OAR3 areas harbor genes with a role in GIN resistance traits (Sweeney et al., 2016).

Finally, the region surrounding marker s16493.1 on OAR6 (Table 1) had no annotated genes and no information on possible functional pathways underlying the control of lnFEC was obtained. In any case, marker s16493.1 is located in the vicinity of segment N of OAR6 on which several genes (mainly belonging to the toll-like receptor and chemokine C-X-C motif ligand families) considered putative candidates to act on GIN resistance have been identified (Benavides et al., 2016).

Furthermore, the current research suggests that OAR2 may play a major role in GIN resistance traits. Our results are highly consistent with the previous identification of two different SNP clusters on OAR2 influencing average FEC in Red Maasai × Dorper sheep (Benavides et al., 2015). Although no genes were annotated in the surroundings of two out the four SNPs associated with GIN resistance traits on OAR2, these markers are located on two different chromosomal areas (from position 49 877 948 to position 64 824 262 and from 117 867 801 to 140 684 314) that deserve future attention. Failing to identify candidate genes in the surroundings of the chromosomal areas associated with the traits studied is not surprising. Bahbahani et al. (2018) consider that “gene deserts” may carry unannotated regulatory elements and are strong candidates for further research.

### 4.2 Candidate genes located close to associated markers

There is scientific consensus on the quantitative nature of GIN resistance (Bishop, 2012; Benavides et al., 2016). Literature is clear in considering that the genetic basis of GIN resistance traits is not only related to genes involved in immune response and acquired immunity but also to genes involved in the gastrointestinal mucus production, parasite expulsion, and hemostasis regulation (Benavides et al., 2016; Sweeney et al., 2016; Zvinorova et al., 2016). In this scenario, the identification of candidate genes with major effects on performance is unlikely and literature gives evidence on multiple potential candidate genes affecting GIN resistance.

The functional cluster for GIN resistance identified in the current research (Table 2), including genes located on OARs 3, 12, 13, and 17, is consistent with expectations. In a meta-analysis, Sayre and Harris (2011) identified up to 14 functional pathways which were common to QTL and gene expression studies for GIN resistance in domestic sheep. Among them, the upregulated pathways were related to immune functions and responsiveness to external signals, while the downregulated pathways were related to cell activity, immune function, and disease. The *TRIB3* gene, on OAR13, encodes a pseudokinase which is upregulated in macrophages and suppresses cytokine expression (Ord and Ord, 2017). The *CDK4* gene, on OAR3, encodes a protein belonging to the serine/threonine kinase family acting on cell proliferation during the cell cycle G1 phase (Sherr et al., 2016). Encoding a protein belonging to the same kinase family, the *CSNK2A1* gene on OAR13, is involved in cell cycle control and apoptosis (St-Denis and Litchfield, 2009). The *MARK1* gene, on OAR12, encodes a kinase involved in the regulation of cell shape and polarity during differentiation, chromosome partitioning at mitosis via the phosphorylation of microtubule-associated proteins (Drewes et al., 1997). Finally, the *SPATA5* gene, on OAR17, encodes an ATPase enzyme involved in cellular development processes and the maintenance of mitochondrial integrity and function (Tanaka et al., 2015).

It is worth discussing the consistency of our findings with previous QTL information available in the literature. Most SNPs overlapping seven previously reported QTLs for GIN resistance traits did not span candidate genes in their surrounding areas. However, two markers associated with FAMACHA scores overlapped with previously reported QTLs for hematocrit (Silva et al., 2012) and immunoglobulin A levels (Atlija et al., 2016) neighboring the *ADAMTS17* (OAR18_5508052_X.1) and the *MBL2* genes (OAR22_6293170.1), respectively. The *MBL2* gene encodes a serum lectin involved in innate host defense (Altorjay et al., 2010). This would confirm the function of the QTL for immunoglobulin A levels reported by Atlija et al. (2016) on OAR22. In turn, the *ADAMTS17* gene encodes a metalloprotease with a role in extracellular matrix degradation and involved in stature in humans (Le Goff and Cormier-Daire, 2011) with no clear relationship with the QTL reported for hematocrit by Silva et al. (2012). In this respect, it is worth mentioning that most QTLs overlapping the regions surrounding the SNPs associated in Djallonké sheep with GIN resistance traits were mainly involved in growth and size (see Table S4). This is not surprising according to the literature: a significant number of QTLs identified for GIN resistance traits seems to be also related to other performance traits (Sweeney et al., 2016). It is well known that, phenotypically, live weight and growth gain are highly correlated with GIN resistance in lambs (Bishop, 2012; Traoré et al., 2017). Although EBVs used as phenotypes in the current research were estimated including live weight as a covariate in the model fitted for additive genetic analyses (Álvarez et al., 2018), an influence of the genes involved in growth and size on GIN resistance are still likely to exist, at least for OAR18 (*ADAMTS17* gene; Le Goff and Cormier-Daire, 2011). Whether a QTL associated with both GIN resistance and performance traits results from pleiotropy or the existence of various loci in close linkage remains unascertained.

## 5 Conclusions

In this research 22 novel genomic areas of putative importance for GIN resistance have been identified in 14 different *Ovis aries* chromosomes. Some of these areas harbored candidate genes involved in immunity-related and cell proliferation processes (mainly genes *TRIB3* and *CSNK2A1* on OAR18, *CDK4* on OAR16, *MARK1* on OAR17, and *SPATA5* on OAR12) of functional importance for the traits analyzed. Furthermore, new insights into the importance of both OAR2 and candidate genes involved in growth have been obtained. This research confirms the importance of obtaining information from local sheep populations managed in harsh environments to gather information on genomic areas of functional importance for GIN resistance.

## Supplement

10.5194/aab-62-313-2019-supplement The supplement related to this article is available online at: https://doi.org/10.5194/aab-62-313-2019-supplement.

## Data Availability

Data used can be made available from the corresponding author on reasonable request after obtaining the permission of all researchers involved in projects AGL2016-77813-R and I1B/4718-1.
