# Supplementary material for: Identification of genomic regions and candidate genes of functional importance for gastrointestinal parasite resistance traits in Djallonké sheep of Burkina Faso
Source: Arch Anim Breed. 2019 Jun 5;62(1):313–23. doi: 10.5194/aab-62-313-2019 (PMC6853132; doi:10.5194/aab-62-313-2019)

Supplement of Arch. Anim. Breed., 62, 313–323, 2019
https://doi.org/10.5194/aab-62-313-2019-supplement
© Author(s) 2019. This work is distributed under
the Creative Commons Attribution 4.0 License.

Open Access
Archives Animal Breeding

Supplement of

Identification of genomic regions and candidate genes of functional importance for gastrointestinal parasite resistance traits in Djallonké sheep of Burkina Faso

Isabel Álvarez et al.

Correspondence to: Félix Goyache (fgoyache@serida.org)

The copyright of individual parts of the supplement might differ from the CC BY 4.0 License.

Table S1. Diversity parameters: mean and standard deviations (in brackets) corresponding to the EBVs estimated for PCV, lnFEC and FAMACHA scores used as phenotypes for association analyses and for observed homozygosity (F). Results are given for the whole dataset and for each of the clusters identified using the software Admixture v1.23. N is the sample size. Additionally between-clusters FST values, computed using the software Arlequin 3.5, are given. All pairwise F_{ST} values were statistically significant for $p < 0.00001$.

cluster	N	PCV	lnFEC	FAMACHA	F	Pairwise F_{ST}			
1	45	1.704 (1.004)	0.251 (0.299)	0.117 (0.291)	0.408 (0.038)				
2	15	2.114 (0.816)	0.475 (0.301)	0.355 (0.301)	0.449 (0.048)	0.103			
3	100	1.285 (1.038)	0.311 (0.436)	0.276 (0.432)	0.37 (0.031)	0.164	0.077		
4	10	2.543 (1.219)	0.212 (0.400)	- 0.169 (0.362)	0.372 (0.052)	0.075	0.017	0.073	
5	14	0.053 (0.720)	- 0.032 (0.333)	- 0.114 (0.373)	0.372 (0.047)	0.138	0.077	0.144	0.064
Whole dataset	184	1.430 (1.126)	0.278 (0.378)	0.189 (0.408)	0.386 (0.044)	1	2	2	4

Table S2. Genes identified within the 125 kb regions surrounding the SNPs associated with PCV, lnFEC and FAMACHA EBVs in Djallonké sheep. The numbers attached to the SNPs (in brackets) and the abbreviations of the gene names are consistent with those listed in Table 1. The *Ovis Aries* chromosome (OAR) number on which the SNP is located is given in brackets when this information is not provided by the SNP identification.

SNP	Gene Start	Gene Stop	ENSEMBL_GENE_ID	Gene Name
(2) s23524.1 (OAR2)	49,732,938	49,799,978	ENSOARG00000011271	tropomodulin 1 (*TMOD1*)
	49,830,043	49,905,937	ENSOARG00000011287	tudor domain containing 7 (*TDRD7*)
(3) OAR2_117867801.1	117,678,117	117,769,907	ENSOARG00000016246	major facilitator superfamily domain containing 6 (*MFSD6*)
	117,796,604	117,807,337	ENSOARG00000016259	inositol polyphosphate-1-phosphatase (*INPP1*)
	117,846,814	117,955,499	ENSOARG00000016273	3-hydroxyisobutyryl-CoA hydrolase (*HIBCH*)
	117,961,639	117,961,923	ENSOARG00000019590	chromosome 2 open reading frame, human C2orf88 (*C2H2orf88*)
(9) s43307.1 (OAR7)	7,209,568	7,373,254	ENSOARG00000016846	synaptic vesicle glycoprotein 2C (*SV2C*)
	7,424,541	7,731,473	ENSOARG00000016888	IQ motif containing GTPase activating protein 2 (*IQGAP2*)
(12) OAR17_34531123_X.1	34,113,507	34,661,626	ENSOARG00000017222	spermatogenesis associated 5 (*SPATA5*)
	34,461,385	34,517,413	ENSOARG00000017455	nudix hydrolase 6 (*NUDT6*)
(15) OAR3_77774489.1	77,735,783	77,736,463	ENSOARG00000016196	ATPase H+ transporting V1 subunit E2 (*ATP6V1E2*)
	77,770,567	77,775,012	ENSOARG00000005641	transmembrane protein 247 (*TMEM247*)
	77,880,264	77,921,519	ENSOARG00000005722	endothelial PAS domain protein 1 (*EPAS1*)
(16) OAR3_161498140.1	161,423,786	161,442,792	ENSOARG00000004820	ATP23 metallopeptidase and ATP synthase assembly factor homolog (*ATP23*)
	161,537,412	161,556,363	ENSOARG00000004836	CTD small phosphatase 2 (*CTDSP2*)

	161,563,274	161,579,047	ENSOARG00000004997	advillin (*AVIL*)
	161,580,697	161,588,504	ENSOARG00000005095	Ts translation elongation factor, mitochondrial (*TSFM*)
	161,590,355	161,596,353	ENSOARG00000005186	methyltransferase like 21B (*METTL21B*)
	161,596,933	161,600,320	ENSOARG00000005201	methyltransferase like 1 (*METTL1*)
	161,601,860	161,606,397	ENSOARG00000005222	25-hydroxyvitamin D-1 alpha hydroxylase, mitochondrial (*LOC101116039*)
	161,610,573	161,612,208	ENSOARG00000005241	membrane associated ring-CH-type finger 9 (*MARCH9*)
	161,617,799	161,623,974	ENSOARG00000005320	cyclin dependent kinase 4 (*CDK4*)
	161,620,781	161,623,542	ENSOARG00000005334	tetraspanin 31 (*TSPAN31*)
(17) OAR12_22189408.1	22,148,091	22,285,882	ENSOARG00000012956	microtubule affinity regulating kinase 1 (*MARK1*)
	22,303,531	22,313,290	ENSOARG00000013070	chromosome 12 open reading frame, human C1orf115 (*C12H1orf115*)
(18) s32476.1 (OAR13)	59,833,973	59,857,609	ENSOARG00000019053	casein kinase 2 alpha 1 (*CSNK2A1*)
	59,881,816	59,890,069	ENSOARG00000019076	TBC1 domain family member 20 (*TBC1D20*)
	59,897,458	59,914,484	ENSOARG00000019106	RANBP2-type and C3HC4-type zinc finger containing 1 (*RBCK1*)
	59,928,054	59,936,548	ENSOARG00000019129	tribbles pseudokinase 3 (*TRIB3*)
	59,961,799	59,968,480	ENSOARG00000019158	neurensin 2 (*NRSN2*)
	60,008,046	60,009,340	ENSOARG00000019161	zinc finger CCHC-type containing 3 (*ZCCHC3*)
	60,017,650	60,037,398	ENSOARG00000019169	chromosome 13 open reading frame, human C20orf96 (*C13H20orf96*)
(20) OAR18_5508052_X.1	5,441,007	5,827,788	ENSOARG00000009766	ADAM metallopeptidase with thrombospondin type 1 motif 17 (*ADAMTS17*)
(21) OAR22_6293170.1	6,177,045	6,182,234	ENSOARG00000013530	mannose binding lectin 2 (*MBL2*)

Table S3. Functional terms enriched clusters identified on the candidate genes identified within the 125 kb regions surrounding the SNPs associated with PCV, lnFEC and FAMACHA scores in Djallonké sheep.

**Annotation Cluster 1**	**Enrichment Score: 1.3021836914741935**											
Category	Term	Count	%	PValue	Genes	List Total	Pop Hits	Pop Total	Fold Enrichment	Bonferroni	Benjamini	FDR
SMART	SM00220:S_TKc	4	11.765	0.017	ENSOARG00000019129, ENSOARG00000005320, ENSOARG00000019053, ENSOARG00000012956	15	328	8147	6.624	0.277	0.277	12.000
INTERPRO	IPR000719:Protein kinase, catalytic domain	4	11.765	0.042	ENSOARG00000019129, ENSOARG00000005320, ENSOARG00000019053, ENSOARG00000012956	28	451	15550	4.926	0.969	0.969	36.873
INTERPRO	IPR011009:Protein kinase-like domain	4	11.765	0.052	ENSOARG00000019129, ENSOARG00000005320, ENSOARG00000019053, ENSOARG00000012956	28	492	15550	4.515	0.987	0.884	43.634
GOTERM_MF_DIRECT	GO:0005524~ATP binding	5	14.706	0.164	ENSOARG00000019129, ENSOARG00000005320, ENSOARG00000017222, ENSOARG00000019053, ENSOARG00000012956	21	1137	10488	2.196	0.984	0.873	75.931

**Annotation Cluster 2**	**Enrichment Score: 0.8579917342705197**											
Category	Term	Count	%	PValue	Genes	List Total	Pop Hits	Pop Total	Fold Enrichment	Bonferroni	Benjamini	FDR
GOTERM_MF_DIRECT	GO:0004674~protein serine/threonine kinase activity	3	8.824	0.048	ENSOARG00000005320, ENSOARG00000019053, ENSOARG00000012956	21	186	10488	8.055	0.679	0.679	32.412
INTERPRO	IPR008271:Serine/threonine-protein kinase, active site	3	8.824	0.080	ENSOARG00000005320, ENSOARG00000019053, ENSOARG00000012956	28	272	15550	6.125	0.999	0.893	58.972
INTERPRO	IPR017441:Protein kinase, ATP binding site	3	8.824	0.092	ENSOARG00000005320, ENSOARG00000019053, ENSOARG00000012956	28	295	15550	5.648	1.000	0.856	64.299
UP_KEYWORDS	ATP-binding	3	8.824	0.326	ENSOARG00000005320, ENSOARG00000019053, ENSOARG00000012956	32	623	16570	2.493	1.000	1.000	96.721
UP_KEYWORDS	Nucleotide-binding	3	8.824	0.439	ENSOARG00000005320, ENSOARG00000019053, ENSOARG00000012956	32	791	16570	1.964	1.000	0.998	99.335

**Annotation Cluster 3**	**Enrichment Score: 0.09438245960976856**											
Category	Term	Count	%	PValue	Genes	List Total	Pop Hits	Pop Total	Fold Enrichment	Bonferroni	Benjamini	FDR
UP_KEYWORDS	Membrane	8	2.353	0.738	ENSOARG00000005334, ENSOARG00000005641, ENSOARG00000019158, ENSOARG00000005320, ENSOARG00000019076, ENSOARG00000005241, ENSOARG00000016846, ENSOARG00000016246	32	4334	16570	0.956	1.000	1.000	99.999
GOTERM_CC_DIRECT	GO:0016021~integral component of membrane	6	1.765	0.805	ENSOARG00000005334, ENSOARG00000005641, ENSOARG00000019158, ENSOARG00000005241, ENSOARG00000016846, ENSOARG00000016246	23	3797	13162	0.904	1.000	1.000	100.000

UP_KEYWORDS	Transmembrane helix	7	2.059	0.839	ENSOARG00000005334, ENSOARG00000005641, ENSOARG00000019158, ENSOARG00000019076, ENSOARG00000005241, ENSOARG00000016846, ENSOARG00000016246	32	4225	16570	0.858	1.000	1.000	100.000
UP_KEYWORDS	Transmembrane	7	2.059	0.840	ENSOARG00000005334, ENSOARG00000005641, ENSOARG00000019158, ENSOARG00000019076, ENSOARG00000005241, ENSOARG00000016846, ENSOARG00000016246	32	4229	16570	0.857	1.000	1.000	100.000

Table S4. Summary of Quantitative Trait Loci (QTL) previously identified in the literature which overlapped the 125 kb regions surrounding the SNPs associated with PCV, lnFEC and FAMACHA score EBVs in Djallonké sheep. Numbers attached to the identifier of the SNP (in brackets) are consistent with those listed in Table 1. QTLs related with the traits analyzed in the present study are in bold.

	QTL				Associated SNP		
OAR	Start	Stop	Description	Source	PCV	lnFEC	FAMACHA
1	3077366	184644010	Muscle weight in carcass QTL	Cavanagh et al., 2010	(1) OAR1_55820164.1		
	9433751	197490407	Lean meat yield percentage QTL	Cavanagh et al., 2010	(1) OAR1_55820164.1		
	10479903	266471112	Carcass fat percentage QTL	Cavanagh et al., 2010	(1) OAR1_55820164.1		
	33739762	239522059	Bone weight in carcass QTL	Cavanagh et al., 2010	(1) OAR1_55820164.1		
	38333681	242147178	Meat eicosapentaenoic acid content QTL	Karamichou et al., 2006	(1) OAR1_55820164.1		
	38333681	249069867	Meat docosapentaenoic acid content QTL	Karamichou et al., 2006	(1) OAR1_55820164.1		
	39019536	62674038	Carcass bone percentage QTL	Cavanagh et al., 2010	(1) OAR1_55820164.1		
	40961414	86433612	Body weight (8 weeks) QTL	McRae et al., 2005	(1) OAR1_55820164.1		
	43893827	249069867	Meat polyunsaturated fatty acid content QTL	Karamichou et al., 2006	(1) OAR1_55820164.1		
2	2206513	206199220	Meat arachidonic acid content QTL	Karamichou et al., 2006	(2) s23524.1; (3) OAR2_117867801.1	(7) OAR2_140684314.1	(14) OAR2_64824262.1
	5842083	31830970	Somatic Cell Score QTL	Raadsma et al., 2009			(21) OAR22_6293170.1
	6946243	238103316	Meat linolenic acid content QTL	Karamichou et al., 2006	(2) s23524.1; (3) OAR2_117867801.1	(7) OAR2_140684314.1	(14) OAR2_64824262.1
	6946243	238103316	Meat eicosapentaenoic acid content QTL	Karamichou et al., 2006	(2) s23524.1; (3) OAR2_117867801.1	(7) OAR2_140684314.1	(14) OAR2_64824262.1
	6946243	234590826	Meat docosapentaenoic acid content QTL	Karamichou et al., 2006	(2) s23524.1; (3) OAR2_117867801.1	(7) OAR2_140684314.1	(14) OAR2_64824262.1
	8804882	248905321	Milk fat percentage QTL	Gutiérrez-Gil et al., 2009a	(2) s23524.1, (3) OAR2_117867801.1	(7) OAR2_140684314.1	(14) OAR2_64824262.1
	25943609	50366707	Rump width QTL	Gutiérrez-Gil et al., 2011	(2) s23524.1		
	32023745	207420807	Milk protein percentage QTL	García-Gámez et al., 2013.	(2) s23524.1; (3) OAR2_117867801.1	(7) OAR2_140684314.1	(14) OAR2_64824262.1
	47450537	65017658	Milk fat yield QTL	García-Gámez et al., 2013.	(2) s23524.1		(14) OAR2_64824262.1
	51986915	65017658	Milk Yield QTL	García-Gámez et al., 2013.			(14) OAR2_64824262.1
	55015296	247088424	Hot carcass weight QTL	Cavanagh et al., 2010	(3) OAR2_117867801.1	(7) OAR2_140684314.1	(14) OAR2_64824262.1
	**61738317**	**137936971**	**Trichostrongylus adult and larva count QTL**	**Crawford et al., 2006**	**(3) OAR2_117867801.1**		(14) OAR2_64824262.1
	77441736	172200261	Ultimate pH QTL	Johnson et al., 2005	(3) OAR2_117867801.1	(7) OAR2_140684314.1	
	77441736	172200261	Meat color a* QTL	Johnson et al., 2005	(3) OAR2_117867801.1	(7) OAR2_140684314.1	
	81462991	158528966	Meat color L* QTL	Johnson et al., 2005	(3) OAR2_117867801.1	(7) OAR2_140684314.1	
	83316173	172200261	Meat color b* QTL	Johnson et al., 2005	(3) OAR2_117867801.1	(7) OAR2_140684314.1	
	83316173	172200261	Meat color a* QTL	Johnson et al., 2005	(3) OAR2_117867801.1	(7) OAR2_140684314.1	
	83316173	172200261	Meat color b* QTL	Johnson et al., 2005	(3) OAR2_117867801.1	(7) OAR2_140684314.1	
	91740470	172200261	shear force QTL	Johnson et al., 2005	(3) OAR2_117867801.1	(7) OAR2_140684314.1	
	92530425	172200261	Meat color L* QTL	Johnson et al., 2005	(3) OAR2_117867801.1	(7) OAR2_140684314.1	
	129310234	203513374	milk lactose yield QTL	Raadsma et al., 2009		(7) OAR2_140684314.1	
3	1184337	224283230	Staple length QTL	Ponz et al., 2001			(15) OAR3_77774489.1; (16) OAR3_161498140.1

	26496578	246140478	Body weight (slaughter) QTL	Cavanagh et al., 2010	(2) s23524.1; (3) OAR2_117867801.1	(7) OAR2_140684314.1	(14) OAR2_64824262.1
	42045510	188726319	internal fat amount QTL	Cavanagh et al., 2010			(15) OAR3_77774489.1; (16) OAR3_161498140.1
	49221453	156721993	Meat conjugated linoleic acid content QTL	Karamichou et al., 2006			(15) OAR3_77774489.1
	131842853	167211326	Body weight (birth) QTL	Roldan et al., 2010			(16) OAR3_161498140.1
	134528475	194352076	Milk fat percentage QTL	García-Gámez et al., 2013.			(16) OAR3_161498140.1
7	2456803	15255352	teat placement QTL	Gutiérrez-Gil et al., 2008		(9) s43307.1	
	2456803	98910176	Staple length QTL	Ponz et al., 2001		(9) s43307.1	
	2456803	98910176	Primary fiber diameter coefficient of variance QTL	Ponz et al., 2001		(9) s43307.1	
	2456803	32151479	Longissimus muscle area QTL	Cavanagh et al., 2010		(9) s43307.1	
**8**	**37510**	**12848380**	**Fecal egg count QTL**	**Atlija et al., 2016.**		**(10) OAR8_8982479.1**	
	**3058289**	**87356552**	**Trichostrongylus adult and larva count QTL**	**Crawford et al., 2006**	**(4) OAR8_16568165.1**	**(10) OAR8_8982479.1**	
	**3058289**	**87356552**	**Trichostrongylus adult and larva count QTL**	**Crawford et al., 2006**	**(4) OAR8_16568165.1**	**(10) OAR8_8982479.1**	
	3058289	79769399	internal fat amount QTL	Cavanagh et al., 2010	(4) OAR8_16568165.1	(10) OAR8_8982479.1	
12	18145496	37948018	Milk Yield QTL	Mateescu and Thonney, 2010			(17) OAR12_22189408.1
13	4100389	67044260	muscle weight in carcass QTL	Cavanagh et al., 2010			(18) s32476.1
	**67497537**	**78896006**	**Fecal egg count QTL**	**Silva et al., 2012**			**(19) s09612.1**
	75498647	81506987	Total fat area QTL	Matika et al., 2016			(19) s09612.1
17	15788973	42385522	Milk Yield QTL	García-Gámez et al., 2013.			(12) OAR17_34531123_X.1
18	1895522	63629707	Testes weight QTL	Fullard et al., 2006	(6) OAR18_43101149.1		(20) OAR18_5508052_X.1
	1895522	63629707	Testes weight QTL	Fullard et al., 2006	(6) OAR18_43101149.1		(20) OAR18_5508052_X.1
	**1895522**	**10588043**	**Hematocrit QTL**	**Silva et al., 2012**			**(20) OAR18_5508052_X.1**
	9943363	68604602	Staple length QTL	Allain et al., 2006	(6) OAR18_43101149.1		
	10964106	62018008	Meat gadoleic acid content QTL	Karamichou et al., 2006	(6) OAR18_43101149.1		
	12038572	63092474	Meat docosahexaenoic acid content QTL	Karamichou et al., 2006	(6) OAR18_43101149.1		
	35128084	47756602	Carcass fat percentage QTL	Cavanagh et al., 2010	(6) OAR18_43101149.1		
22	259746	6415933	muscle weight in carcass QTL	Matika et al., 2016			(21) OAR22_6293170.1
	259746	6415933	Carcass bone percentage QTL	Matika et al., 2016			(21) OAR22_6293170.1
	259746	6415933	Body weight (slaughter) QTL	Matika et al., 2016			(21) OAR22_6293170.1
	259746	6415933	Hot carcass weight QTL	Matika et al., 2016			(21) OAR22_6293170.1
	4058030	8888071	bone density QTL	Matika et al., 2016			(21) OAR22_6293170.1
	**5331079**	**7374882**	**Immunoglobulin A level QTL**	**Atlija et al., 2016.**			**(21) OAR22_6293170.1**

Figure S1. Plot showing cross-validation errors for each K tested using the software Admixture v1.23. The lower error was found for K = 5 and, therefore, this would be the optimal number of clusters in data.

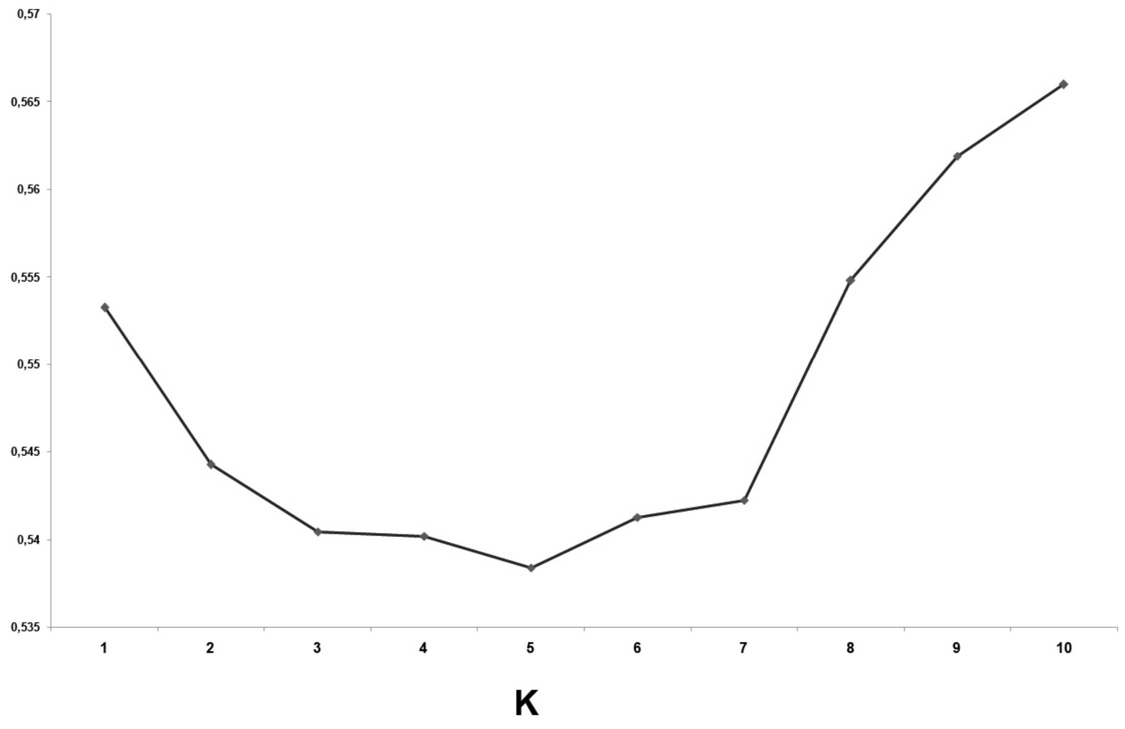

Supplement: The supplement related to this article is available online at: https://doi.org/10.5194/aab-62-313-2019-supplement. [file aab-62-313-supplement.pdf]
